# A hyaluronic acid- and chondroitin sulfate-based medical device improves gastritis pain, discomfort, and endoscopic features

**DOI:** 10.1007/s13346-018-0531-7

**Published:** 2018-05-23

**Authors:** Tommaso Iannitti, Julio César Morales-Medina, Alberto Merighi, Valentina Boarino, Carmen Laurino, Maria Vadalà, Beniamino Palmieri

**Affiliations:** 1 KWS BioTest, 47-48 Martingale Way, Marine View Office Park, BS20 7AW Portishead, Somerset UK; 2 Centro de Investigación en Reproducción Animal, CINVESTAV-Universidad Autónoma de Tlaxcala, AP 62, CP 90000 Tlaxcala, Mexico; 3 Department of Gastroenterology, Division of Digestive Endoscopy, University of Modena and Reggio Emilia, 41124 Modena, Italy; 4 Department of General Surgery and Surgical Specialties, University of Modena and Reggio Emilia Medical School, Surgical Clinic, 41124 Modena, Italy; 5 Second Opinion Medical Network, Modena, Italy

**Keywords:** Gastritis, Chondroitin sulfate, Hyaluronic acid, Endoscopy, Pain, Discomfort

## Abstract

Gastritis is an inflammation of the gastric mucosa. In this study, we investigated the efficacy of a medical device, Esoxx®, based on hyaluronic acid and chondroitin sulfate on gastritis-related upper abdominal pain/discomfort and endoscopic features. Fifty patients, affected by gastritis, were randomised to receive the medical device or placebo. The primary endpoint was the medical device efficacy on upper abdominal pain/discomfort associated with gastritis and measured by Visual Analogue Scale (VAS). The secondary endpoints were the efficacy of the medical device on gastritis-related mucosal erosions, blood oozing, and hyperemia (redness)/edema, as assessed by endoscopy, and the patients’ rating of their compliance with the treatments. A significant reduction in VAS pain was observed in the treatment group after a 5-week treatment, if compared with placebo (*p* < 0.001). In summary, administration of a medical device, based on hyaluronic acid and chondroitin sulfate, improves gastritis-related upper abdominal pain/discomfort and decreases mucosal erosions, blood oozing, and hyperemia (redness)/edema at 5-week follow-up in patients affected by gastritis.

**Electronic supplementary material:**

The online version of this article (10.1007/s13346-018-0531-7) contains supplementary material, which is available to authorized users.

## Introduction

Gastritis is commonly defined as a histologically confirmed inflammation of the gastric mucosa and affects up to 50% of the population worldwide [1]. Gastritis can be triggered by multiple factors including *Helicobacter pylori* infection, biliary reflux into the stomach, use of non-steroidal anti-inflammatory drugs, unbalanced diet, chemical injuries such as alcohol and acids, and long-term physical and mental stress [1, 2]. This inflammatory condition can result in mucosal erosions, blood oozing, and hyperemia (redness)/edema with inflammatory cell infiltration of the gastric layers [3–5]. The symptomatic treatment of gastritis can be managed by proton pump inhibitors to reduce acid output and buffering products that can counteract the hydrogen ion-induced damage to the mucosa. Glycosaminoglycans, including chondroitin sulfate (CS) and hyaluronic acid (HA), are expressed in human gastric tissue [6]. Sulfated glycosaminoglycans, chondroitin 4,6-sulfate, dermatan sulfate and heparan sulfate have been observed in two gastric regions, the antrum and the body of the stomach, in patients affected by chronic superficial gastritis [7].

CS is a member of the glycosaminoglycan family and, in vertebrates, consists of repeating sulfate-substituted GalNAcβ4GlcAβ3 disaccharide units polymerised into long chains [8]. CS molecular structure was identified by Babkin and Komarov [9] as an effective inhibitor of pepsin-induced damage to the gastroduodenal mucosa. Pepsin, together with mucoitinsulfate, is a key chemical component of the mucous that is spontaneously secreted by the parietal cells. CS has been extensively used for treatment of symptomatic knee osteoarthritis improving pain and overall mobility and has showed structure-modifying effects in knee and finger osteoarthritis [10]. Furthermore, CS has shown good tolerability and safety in the clinical setting [10] and potent anti-inflammatory properties in animal models of arthritis [11]. HA is a non-sulfated, naturally occurring glycosaminoglycan consisting of alternately repeating D-glucuronic acid and N-acetylglucosamine units [12]. HA interacts with several cell surface receptors such as cluster determinant 44 (CD44) and the receptor for hyaluronate-mediated motility (RHAMM), which have been associated with malignant transformation of gastric mucosa, although their expression has also been reported in non-malignant mucosa [13–15]. HA is also involved in innate immune response and inflammation since it participates in leukocyte recruitment via interaction with CD44, activating inflammatory cells, such as macrophages, through CD44-dependent signaling. HA also induces dendritic cell maturation and promotes cytokine release by dendritic cells and endothelial cells through toll-like receptor 4 [16]. Further studies have also shown that HA possesses antibacterial, antifungal [17], and antiviral activities [18]. Due to its biological properties, HA has been extensively used in experimental and clinical osteoarthritis [19–21], lower-leg telangiectasia [22], premature ejaculation [23], and restorative and esthetic surgery [24–26]. However, to date, no clinical study has shown an effectiveness of a compound based on HA and CS on gastritis-related upper abdominal pain/discomfort and endoscopic features. In the present study, we hypothesized that HA and CS would steadily coat the epithelial surface of the gastric mucosa stimulating the healing process in a subset of patients affected by gastritis characterized by upper abdominal pain/discomfort and mucosal erosions, blood oozing, and hyperemia (redness)/edema.

## Materials and methods

### Patients and study design

This retrospective, anecdotal, double-blind randomised placebo-controlled study was conducted in accordance with the Declaration of Helsinki and institutional review board rules.

Fifty patients (females = 18; males = 32; body mass index = 18.5–24.9) aged between 6 and 87 years [50.2 ± 2.3, mean ± standard error of the mean (SEM)] who had appealed to our “Second Opinion Medical Network” (Modena, Italy) between 2016 and 2017 due to gastritis symptoms were included in this study. The concept of “Second Opinion Medical Consulting Network” has been reviewed elsewhere [27–31]. Before the beginning of the study, the patients underwent a complete physical examination. A gastrointestinal endoscopy (criteria for diagnosis of gastritis were bleeding, vascular pattern [congestion], and excess mucous secretion) and gastric biopsy (from antrum or pyloric areas in all forms of gastritis and mainly from the gastric body for chemical gastritis) were carried out to confirm the diagnosis of gastritis. Eighteen patients presented non-atrophic gastritis (possibly due to unbalanced diet and lifestyle), 8 patients were affected by atrophic gastritis and 24 by chemical gastritis (bile reflux observed by endoscopy) based on the classification by Dixon and coworkers [32]. The inclusion criteria for gastritis were epigastric burning, bloatedness, nausea, meteorism, and belching, accompanied by mucosal erosions, blood oozing, hyperemia (redness)/edema, and upper abdominal pain/discomfort ≥ 40 mm, as measured by Visual Analogue Scale (VAS). The patients included in this study also had dyspepsia. All the symptoms described above were present in all the types of gastritis previously mentioned. Patients were divided into two groups made up of 9 females and 16 males each and were randomised to receive either the medical device or placebo. The patients were instructed to stop previous medical prescriptions for treatment of gastritis including proton pump inhibitors, other buffering and gastroprotective agents, and digestive enzymes 7 days before the beginning of the study. Exclusion criteria for the present study were presence of ulcers at any gastric segment, pyloric stenosis, *Helicobacter pylori* infection (ruled out by breath test), esophageal stricture or intestinal obstruction, previous gastrointestinal surgery, and a known hypersensitivity to the compounds object of the present study. Patients who received prolonged non-steroidal anti-inflammatory drug therapy in the year preceding this study were also excluded.

### Treatment

The medical device (Esoxx®, Alfa Wassermann, Bologna, Italy) is based on a mixture of hyaluronic acid and chondroitin sulfate in a bioadhesive carrier Lutrol® F 127 (poloxamer 407; BASF, Milan, Italy) that acts as a buffering agent to form a barrier and prolong the action on the esophageal mucosa [33]. The formulation also contains polyvinylpyrrolidone, xylitol C, sodium benzoate, potassium sorbate, aromas, and demineralized water. Esoxx® has been proposed for the treatment of the symptoms of gastroesophageal reflux disease and produces a persistent mucosal barrier, as shown by ex vivo studies performed in the swine model [34].

The placebo composition was as follows: 10% Vaseline oil/water emulsion, viscosity enhancer, preservatives, aroma, and water. The used formulations were manufactured by Alfa Wasserman Spa (Bologna, Italy). Ten milliliters of the placebo or medical device was administered four times a day (prior to breakfast, lunch, dinner, and bedtime) for 2 weeks. The 2-week treatment was followed by a week without any medication. Afterwards, the patients underwent 2-week further treatment according to the above-described protocol. We used a 5-week design as we hypothesized that this timecourse was necessary to allow long-lasting protection of the mucosa from gastric acid and promote the repair of histological lesions.

### Assessment of upper abdominal pain/discomfort

VAS, a scoring system from 0 (minimum pain) to 100 mm (severe pain), was used to rate the primary endpoint, i.e., improvement in gastritis-related upper abdominal pain/discomfort at 5-week follow-up. In regard to the two children involved in this investigation, their parents were allowed to stay to give their support in relation to the VAS pain scoring. A month after the end of the study, a phone interview was used to determine if the patients’ improvement in gastritis-related upper abdominal pain/discomfort was still persisting.

### Assessment of gastritis-related mucosal erosions, blood oozing, and hyperemia (redness)/edema

The secondary endpoints were evaluation of the effect of the medical device on gastritis-related mucosal erosions, blood oozing, and hyperemia (redness)/edema, compared to placebo, as assessed by photographic endoscopy evaluation performed by two blinded pathologists at 5-week follow-up. The pathologists gave a judgment according to the following ranges: (1) 1–30% = poor improvement, (2) 30–60% = moderate improvement, and (3) 60–100% = good improvement.

### Patients’ compliance and medical device tolerability

The patients were also asked to rate their compliance/tolerability related to viscosity, taste, and difficulty to swallow the treatments as “poor,” “fair,” “good,” or “very good.”

### Statistical analysis

VAS data were analyzed using a two-way analysis of variance (ANOVA) followed by Bonferroni post hoc test using GraphPad Prism (GraphPad Software Inc., San Diego, CA, USA). All data are presented as the means ± SEM. A *p* value ˂ 0.05 was considered significant.

## Results

### Upper abdominal pain/discomfort

**Fig. 1:**
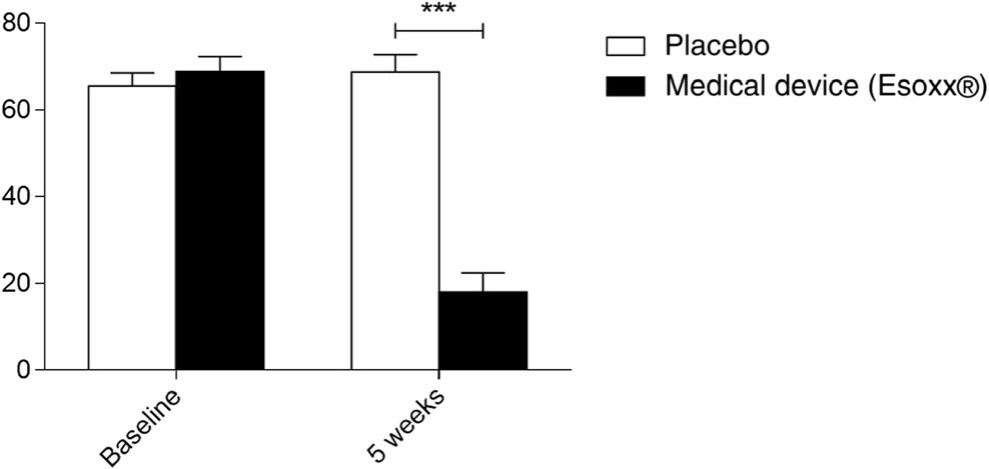
Comparison of gastritis-related upper abdominal pain/discomfort between patients treated with the medical device (*n* = 25) and patients receiving placebo (*n* = 25), as assessed by VAS at 5-week follow-up. Data are reported as the means ± SEM. ***p ˂ 0.001 VAS Visual Analogue Scale, SEM, standard error of the mean

At baseline, upper abdominal pain/discomfort was 65.56 ± 2.9 mm for the placebo group and 68.8 ± 3.4 mm for the treatment group. A significant reduction in upper abdominal pain/discomfort was observed in the treatment group, if compared with placebo at 5-week follow-up (Fig. 1). Among the patients who underwent medical device treatment, 16 patients, including the 2 children, reported high relief from upper abdominal pain/discomfort (post-treatment VAS range = 0–25 mm), 7 reported a moderate reduction in upper abdominal pain/discomfort (post-treatment VAS range = 25–50 mm), and 2 patients presented only a slight reduction in upper abdominal pain/discomfort (post-treatment VAS range = 50–100 mm). All patients in the placebo group fell in the 50–100 mm range showing an improvement in upper abdominal pain/discomfort up to 15%.

At the phone interview, improvement in upper abdominal pain/discomfort was persistent in 23 patients from the medical device group. The 2 patients from this group who did not experience amelioration in upper abdominal pain/discomfort at 5-week follow-up started a different therapy. No amelioration in upper abdominal pain/discomfort in all patients receiving placebo was observed at the phone interview.

### Gastritis-related mucosal erosions, blood oozing, and hyperemia (redness)/edema

**Fig. 2:**
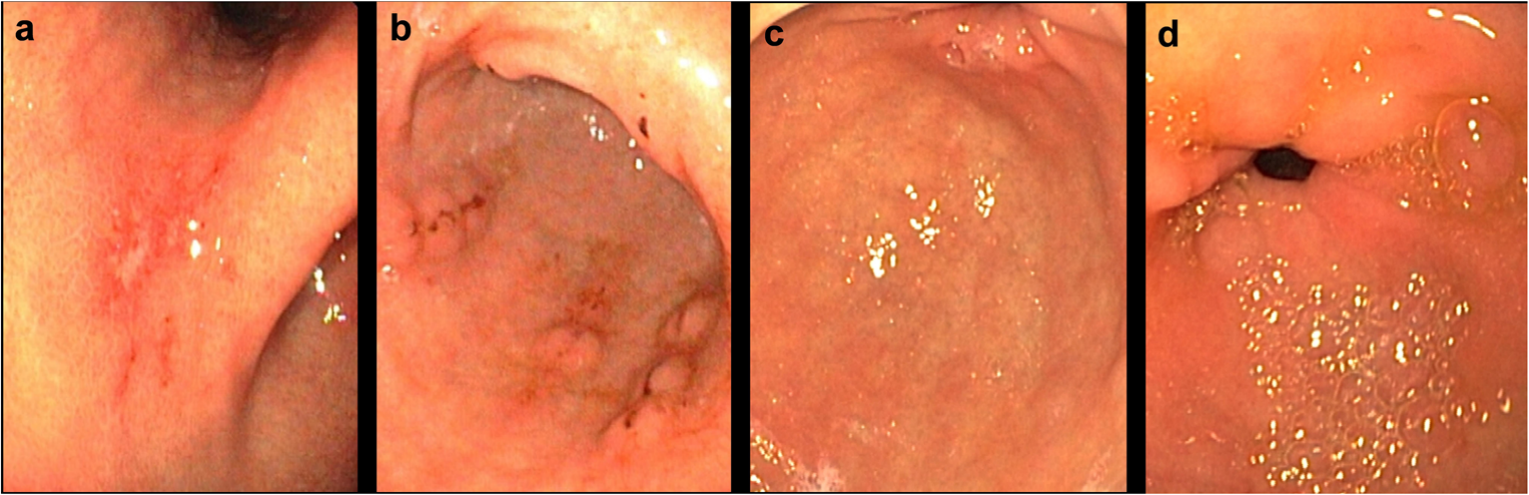
Gastritis at baseline (**a** and **b**) and 5 weeks following medical device administration (**c** and **d**). Gastric erosions with fibrin streaks are visible in (**a**). Gastric erosions with hematin pigments are visible in (**b**). Definite improvement is observed after treatment with the medical device (**c** and **d**)

**Fig. 3:**
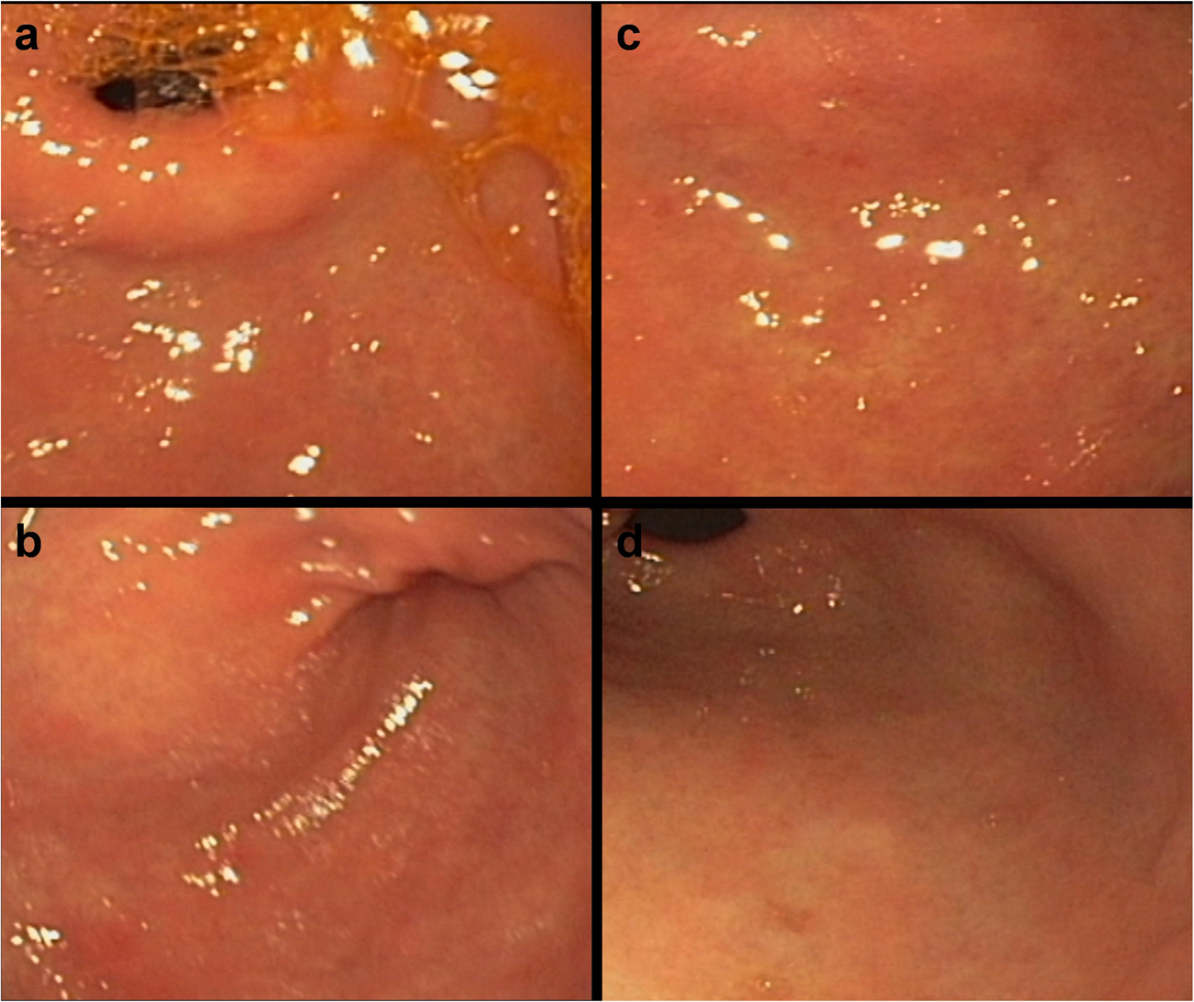
Gastritis at baseline (**a**) and 5 weeks following placebo administration (**c**). Gastritis with reddening and swelling at baseline (**b**) and 5 weeks following placebo administration (**d**). No improvement in gastritis can be observed at the 5-week follow-up (**c** and **d**)

Endoscopic assessment at baseline was compared to the endoscopy performed at 5-week follow-up in terms of erosions, blood oozing, and hyperemia (redness)/edema in the active treatment (Fig. 2) and placebo (Fig. 3) groups. Among the 25 patients who underwent medical device treatment, 17 showed good endoscopic healing according to the above-mentioned parameters, as judged by the two pathologists, 6 showed moderate improvement, and 2 patients showed a poor improvement (these were the same patients who showed only slight improvement in upper abdominal pain/discomfort). The improvement in these parameters was also consistent with amelioration in the dyspeptic symptoms observed at baseline. All patients in the placebo group showed poor improvement in all endoscopic features analysed in this study.

### Patients’ compliance and medical device tolerability

The patients treated with the medical device rated their compliance/tolerability related to viscosity, taste, and difficulty to swallow as good (*n* = 6) and very good (*n* = 19). Twenty-two patients in the placebo group rated their compliance/tolerability as very good, while 3 rated their compliance as good. No adverse effects were observed in both groups.

## Discussion and conclusions

The present study included 50 patients affected by gastritis characterized by upper abdominal pain/discomfort of at least 40 mm, as measured by VAS. A 5-week administration of a medical device based on HA and CS reduced gastritis-related upper abdominal pain/discomfort in 23 patients at 5-week follow-up, if compared with placebo. This improvement persisted at the phone interview performed a month after the end of the study. The reduction in VAS score was coupled with amelioration in blood oozing, hyperemia (redness)/edema, and mucosal erosions, as assessed by two pathologists by photographic endoscopic examination at 5-week follow-up. A first-in-man attempt to treat gastroduodenal diseases by oral administration of CS was performed by Crandall and Roberts [35] on 22 patients affected by duodenal peptic ulcer with a 45% improvement in symptoms. In line with these results, CS promoted healing of skin ulcer in the rat [36]. Furthermore, Harrison and colleagues [37] showed that CS is an excellent coating for intraocular lens implantation in order to avoid damage to the corneal epithelium. According to this study, CS surpassed the protective qualities of other compounds, while albumin was second best and HA third. Furthermore, CS was the most efficacious protective agent with an effect lasting 40 hours, if compared with sodium hyaluronate. The concept of a protective layer made by CS upon the surface of mucosal lesions is very appealing and can be achieved due to the high affinity of the compound for the injured surfaces leading to a very effective and robust protection [38–41]. Another clinical study reported the anti-inflammatory and healing properties of CS showing that intravesical instillation of 0.2% highly purified CS solution (molecular weight = 20–40 Da) in patients with interstitial cystitis showed a favorable symptomatic outcome in this muscular-epithelial contractile organ [42].

The results from the present study strongly support the hypothesis that HA may cover the submucosal connective tissue inducing epithelial cell shifting and increasing cell motility. In turn, this tissue becomes softer and hydrophilic because of HA availability beneath the mucosa containing fibrin and mucous allowing the repair of the damaged gastric mucosa. At the same time, CS may act synergistically to promote, together with HA and the added adhesive biopolymer, the healing of ulcers and erosions. We speculate that the use of this composition may also be extended to manage the symptoms related to esophagitis, gastrointestinal reflux, and other gastroduodenal diseases although this will need to be proven by future clinical studies. Our investigation presents limitations such as the small number of patients and the lack of a long-term endoscopic follow-up to assess the effect of this treatment in the long run. Furthermore, patients presenting *H. pylori*-related gastritis have not been taken into account. In conclusion, we speculate that the effectiveness of Esoxx® may rely on HA and CS ability to coat the gastric epithelium, inhibiting gastric fluid acidity and pepsin-induced mucosal erosion. Further studies, involving larger cohorts of patients, are necessary to establish the long-term efficacy of Esoxx® and its underlying mechanism.

## Electronic supplementary material

ESM 1 (DOCX 2745 kb)
