# Supplementary material for: A hyaluronic acid- and chondroitin sulfate-based medical device improves gastritis pain, discomfort, and endoscopic features
Source: Drug Deliv Transl Res. 2018 May 23;8(5):994–9. doi: 10.1007/s13346-018-0531-7 (PMC6133073; doi:10.1007/s13346-018-0531-7)
Supplement: Supplementary file 1 — (DOCX 2745 kb) [file 13346_2018_531_MOESM1_ESM.docx]

**Fig. 1** – Comparison of gastritis-related upper abdominal pain/discomfort between patients treated with medical device (n = 25) and patients receiving placebo (n = 25), as assessed by VAS at 5-week follow-up. Data are reported as the means ± SEM. *** p ˂ 0.001

VAS, Visual Analogue Scale; SEM, Standard Error of the Mean

**Fig. 2** – Gastritis at baseline (a and b) and 5 weeks following medical device administration (c and d). Gastric erosions with fibrin streaks are visible in figure a. Gastric erosions with hematin pigments are visible in figure b. Definite improvement is observed after treatment.

**Fig. 3** – Gastritis at baseline (a) and 5 weeks following placebo administration (c). Gastritis with reddening and swelling at baseline (b) and 5 weeks following placebo administration (d). No improvement in gastritis can be observed at the 5-week follow-up (c and d).
